# Laser acupuncture and photobiomodulation therapy in Bell’s palsy with a duration of greater than 8 weeks: a randomized controlled trial

**DOI:** 10.1007/s10103-023-03970-4

**Published:** 2024-01-13

**Authors:** Dong Wu, Xin Lan, Gerhard Litscher, Yan-Ling Zhao, Yun-Qing Wu, Ru-Jun Dai, Kai Cao, Yu Wang, Lu-Quan Chen

**Affiliations:** 1 Department of Traditional Chinese Medicine, Beijing Tongren Hospital, Capital Medical University, Beijing, 100730 China; 2 Jococo Inc, Los Angeles, CA USA; 3 President of the International Society for Medical Laser Applications (ISLA transcontinental; since 2012), German Vice President of the German-Chinese Research Foundation (DCFG) for TCM (since 2014), Vice Chairperson, World Federation of Chinese Medicine Societies, Committee of Card. Rehab. (2023-2028), Honorary President of the European Federation of Acupuncture and Moxibustion Societies (2023), Honorary Professor of China Beijing International Acupuncture Training Center, China Academy of Chinese Medical Sciences (2023), Former Head of two Research Units at Medical University of Graz, 8036 Graz, Austria; 4 Department of Otolaryngology Head and Neck Surgery, Beijing Tongren Hospital, Capital Medical University, Beijing, China; 5 Department of Neurology, Beijing Tongren Hospital, Capital Medical University, Beijing, China; 6 TED Healthcare Technology Ltd (Beijing), Beijing, China; 7 Beijing Institute of Ophthalmology, Beijing Tongren Hospital, Capital Medical University, Beijing, China; 8 Institute of Acupuncture-Moxibustion, China Academy of Chinese Medical Sciences, Beijing, China

**Keywords:** Bell’palsy, Laser acupuncture, RCT, ENoG, EMG, Rehabilitation, Photobiomodulation

## Abstract

To investigate the efficacy of laser acupuncture and photobiomodulation therapy in alleviating symptoms among patients diagnosed with Bell’s palsy with duration of greater than 8 weeks. The randomized controlled trial has been performed from May 2021 to April 2023. Patients were eligible who had Bell’s palsy with duration of greater than 8 weeks on out-patient Department of Otorhinolaryngology in Beijing Tongren Hospital. The laser acupuncture group received class IV laser treatment for 3 times per weeks, a total of 72 times. The control group received the same treatment procedure except the laser parameter. The primary outcome measures comprised House–Brackmann facial nerve grading system and electroneurography. Secondary outcome measures comprised Sunnybrook facial grading system, electromyography, and the blink reflex. A total of 84 participants were included (42 control group, 42 laser acupuncture group). After treatment, House–Brackmann facial nerve grading system (OR, 0.11; 95% CI, 0.04–0.30; P < 0.001), and the pathologic numbers of electroneuronography were statistically different between the laser acupuncture group and control group, including orbicularis oculi (OR,0.08; 95% CI, 0.02–0.21; P < 0.001), Frontalis muscle (OR,0.14; 95% CI, 0.05–0.39; P < 0.001), Orbicularis oris (OR,0.13; 95% CI, 0.04–0.36; P < 0.001), Ala nasi muscle (OR,0.06; 95% CI, 0.02–0.18; P < 0.001). In secondary outcomes, Sunnybrook facial grading system, has significant difference between the two groups (20.26; 95% CI, 14.69 to 25.83; P < 0.01). Latency by ENoG, include orbicularis oculi (-0.61; 95% CI, -0.43 to -0.09; P < 0.001), frontalis muscle (-0.12; 95% CI, -0.21 to -0.03; P < 0.01), orbicularis oris (-0.28; 95% CI, -0.41 to -0.16; P < 0.001), and ala nasi muscle (-0.26; 95% CI, -0.38 to -0.16; P < 0.001). All amplitudes of MUAPs and durations by electromyography (EMG) showed statistically significant differences compared with the control group after treatment. For the frontalis muscle, the amplitude of MUAPs was -64.23 (95% CI, -80.89 to -47.56; P < 0.001) and duration was -1.18 (95% CI, -1.49 to -0.87; P < 0.001). For orbicularis oris, amplitude of MUAPs was -29.82 (95% CI, -55.03 to -4.62; P = 0.02) and duration was -0.57 (95% CI, -0.94 to -0.20; P < 0.001). For depressor angulli oris, amplitude of MUAPs was -47.06 (95% CI, -62.15 to -31.97; P < 0.001) and duration was -2.21 (95% CI, -2.69 to -1.72; P < 0.001). Blink reflex, including R1 (OR, 0.03; 95% CI, 0.01–0.16; P < .001), R2 (OR, 0.04; 95% CI, 0.004–0.29; P < .001), and R2 latency differences (OR, 0.15; 95% CI, 0.05–0.51; P < .001), have significant difference between the two groups, respectively. The findings suggest that laser acupuncture relieve symptoms for patients with Bell’s palsy with a duration of greater than 8 weeks.

Trial registration: ClinicalTrials.gov Identifier: NCT05846217.

## Introduction

Bell's palsy can stem from a range of underlying causes, such as viral infections, autoimmune diseases, diabetes mellitus, emotional factors, stress, and iatrogenic factors [1]. This condition results in facial weakness or paralysis, alongside symptoms like impaired or altered taste, hyperacusis, and reduced salivation and tear secretion [2, 3]. Beyond the functional and aesthetic concerns, facial paralysis can obstruct in-person communication and give rise to profound psychological complications [4].

Different treatments have been proposed to achieve rapid recovery without significant sequelae. Such treatments include facial expression exercises [5], corticoids [1], antiviral drugs [6], electrical stimulation [7], and photobiomodulation therapy (PBMT) [8]. Although most patients recover from the functional nerve dysfunction, some patients exhibited an incomplete recovery [9].

PBMT, a non-invasive and cost-effective option with less known adverse effects, encompasses low-level laser therapy and laser acupuncture [10, 11]. The treatment method includes low-level laser therapy and laser acupuncture [12, 13]. Studies indicate that laser acupuncture for Bell's palsy can reduce pain and have anti-inflammatory effects, though the exact mechanism remains unclear [14, 15].

Although the underlying mechanisms of laser in the treatment of facial paralysis are still unclear, several studies suggest that laser acupuncture has been suggested for the treatment of Bell’s palsy demonstrating an immediate pain decrease as well as an anti-inflammatory effect [16, 17]. However, there is a lack of randomized controlled trials validating its efficacy beyond 8 weeks. Many studies are limited to subjective assessments [18, 19].

Acupuncture has been found to be beneficial for Bell's palsy [20, 21], however, there have been no randomized controlled trials conducted to validate its efficacy in Bell’s palsy over 8 weeks. Therefore, the aim of this study was to evaluate the effectiveness of laser acupuncture and photobiomodulation therapy in patients undergoing Bell’s palsy with duration of greater than 8 weeks by subjective scale and electrophysiological testing.

## Materials and Methods

### Study Design and Setting

This single-center, single-blind, randomized controlled trial was conducted from May 1, 2021, to April 10, 2023, at the Outpatient Department of Otorhinolaryngology at Beijing Tongren Hospital, Capital Medical University, Beijing, China. The ethics committee of the Beijing Tongren Hospital, Capital Medical University approved the study (TREC2022-KY075). The study was registered at Clinical-Trials.gov (NCT05846217).

### Inclusion criteria

Patients selected had Bell's Palsy with a duration of greater than 8 weeks. All the patients were diagnosed by the departments of Otolaryngology in Beijing Tongren Hospital. No medications were taken within 2 weeks. Also, patients were eligible if they were graded at House–Brackmann grade (HB) 3 or higher. They were adults over 18 years of age and under 60 years of age; and had not received medications in the 2 weeks prior to the trial, such as prednisolone.

### Exclusion criteria

Patients with HB grade 6 patients, or those with greater than 90% denervation on electroneuronography, or no voluntary electromyography activity, or no latency of early (R1) and late (R2, R2’) components in blink reflex were excluded.

Exclusion criteria also included serious mental illness or social problems, and neurological disorders, and systemic diseases, such as severe diabetes, malignant tumors, and other serious consumptive diseases, as well as those planning for pregnancy, those in pregnancy, or those who were lactating [1]. We also excluded Bell’s Palsy patients who have a disease course of more than 1 year. All participants gave written informed consent before the start of the study.

### Randomization and blinding

Computer-generated randomization lists were done by randomization in blocks of 4, prepared by an independent statistician using SAS software (version 9.4; SAS Institute, Cary, NC), and sequential number was concealed in sealed opaque envelopes. Envelopes were opened only after participants were enrolled. Investigator in our study group enrolled participants and assigned participants to interventions. Study personnel who involved in recruitment and data collection were not involved in clinical management. The study patients and data analysts were blinded to intervention assignment, but the physicians were not blinded.

### Interventions

Patients in the laser acupuncture group (LA group) received 72 sessions of laser acupuncture (3 times per week). Laser acupuncture used a class IV Multiwave Locked System (MLS) laser (Mphi laser, ASA Srl, Vicenza, Italy). MLS laser is a class IV NIR (near infrared) laser with two synchronized sources (905 nm with 75 W peak power, pulsed mode; 808 nm with power 1 W, continuous mode). Both laser beams were synchronized, the locked waves work with the range 1–2000 Hz.

**Fig. 1:**
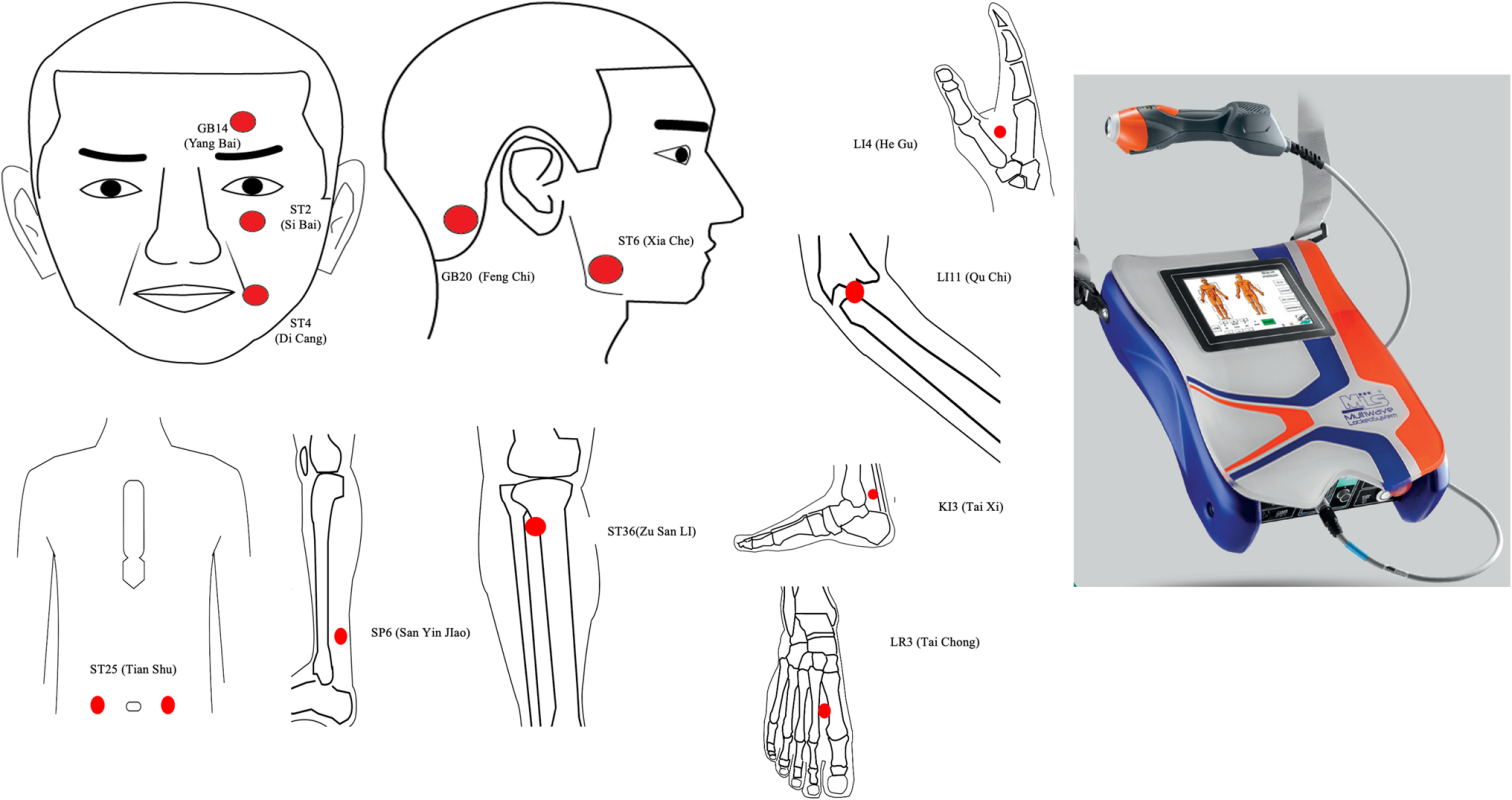
Acupuncture points used in the present study and laser acupuncture equipment

In the LA group, based on clinical experience, we selected 5 acupoints on the affected side, including ST2 (Si Bai), ST4 (Di Cang), ST6 (Jia Che), GB14 (Yang Bai), and GB20 (Feng Chi). Additionally, we chose 7 acupoints: LI4 (He Gu), LI11 (Qu Chi), ST25 (Tian Shu), ST36 (Zu San Li), SP6 (San Yin Jiao), KI3 (Tai Xi), and LR3 (Tai Chong). The acupoints on the limbs and trunk were applied bilaterally. The selection of acupoints is based on previous research and our clinical experience[22, 23] (Fig. 1).

Laser probe directly contacts with skin of the all the acupoints. The probe was fixed on each point for 1 min. In this study, the laser acupuncture used have wavelength of 808 nm and 905 nm, 1.2 W power (808 nm is 1 W, 905 nm is 200 mW), continuous mode emission (808 nm) and pulsed mode emission (905 nm), 1500 Hz, 50% power level, 50% duty cycle, 8.35 J/cm^2^ dosimetry, 26.22 J for each point, administered for 3 times per week, 72 times total treatments. The control group received the same Multiwave Locked System device, same points in affected face and acupoints. However, the laser acupuncture used 1 Hz, 25% power level, 50% duty cycle, 2.63 J/cm^2^ dosimetry, 8.26 J for each point. The treatment in control group was regarded as ineffective. Laser device was calibrated by TED Healthcare Technology Ltd, Beijing, China.

All patients in LA group wore safety glasses to prevent eye damage during the laser sessions. All treatments were performed in the outpatient clinic by the same physician (Table 1).

**Table 1:** Laser Parameters

Device information	Manufacturer	ASA (S.r.l., Vicenza, Italy)	
	Model identifier	MLS laser, Mphi	
	Year produced	2020	
	Number of emitters	1	
	Emitter type	NIR laser with two synchronized laser diodes	
Irradiation parameters	Center wavelength	Laser diode 1	Laser diode 2
		905 nm	808 nm
	Operating mode	Pulsed wave	Continuous wave
	Power	200 mW	1000 mW
	Peak radiant power	75 W	1.0 W
	Frequency range	1–2000 Hz	
	Power level	50%	
	Target area diameter	ø 2 cm	
	Beam profile	Two laser beams work simultaneously and synchronously with coincident propagation axes	
	Application technique	Contact	
	Irradiance or power density	0.19 W/cm^2^	
	Number of points irradiated	5 acupoints in the affected side. 7 acupoints applied bilaterally. A total of 19 acupoints	
	Duration of each treatment session	1140 s	
	Dose of each point	26.22 J	
	Dose in the form of energy density	8.35 J/cm^2^	
	Cumulative dose of each treatment session	498.16 J	
	Frequency of treatment	3 times per weeks	
	Total treatment session	72 times	

### Outcome Measure

Primary Outcome comprised HB, electroneuronography (ENoG). Secondary Outcome Measures comprised Sunnybrook Facial Grading Scale (SB Grading) and latency by ENoG, electromyography (EMG) and Blink Reflex. All outcome measures were conducted on the 1st and 180th days after informed consent were obtained.

Primary Outcome comprised HB, electroneuronography (ENoG). Secondary Outcome Measures comprised Sunnybrook Facial Grading Scale (SB Grading) and latency by ENoG, electromyography (EMG) and Blink Reflex. All outcome measures were conducted on the baseline and 180th days after informed consent were obtained.

HB was employed to evaluate the facial motor function [24]. The prognoses of grade 3 or higher were abnormal. All the HB grading were assessed by the same medical chief physician. SB Grading is 13-items questionnaire that used to evaluate the facial movement of patients [25]. All the operations of HB and SB Grading by the same chief physician.

ENoG and EMG are now the most important facial electrophysiological examinations [26, 27]. ENoG involves recording the compound muscle action potentials (CMAPs) and latencies of muscles, including orbicularis oculi, frontalis muscle, orbicularis oris, and ala nasi muscle [28, 29]. A percentage of degenerated nerve fibers is calculated by the amplitude of the CMAPs, a side difference of 30% or greater is considered pathologic [26]. EMG is an electrophysiologic measures by recording motor unit action potentials (MUAPs) in the muscle of depressor angulli oris, frontalis muscle and orbicularis oris. The larger the value, the more severe the facial nerve damage [30]. The Blink reflex test is used to measure the facial nerve since the blink reflex delivers information on facial nerve function with normal trigeminal function[26].

In Blink reflex testing, two responses, R1 and R2, are analyzed. R1 is the fast ipsilateral response of the orbicularis oculi muscle with a latency of about 10–12 ms. The second bilateral response R2 has a latency of about 30–41 ms. The R1 latency of higher than 12 ms, or the R2 latency of higher than 41 ms is considered pathologic. The R2 latency differences between both sides greater than 8 ms are considered pathologic.

Dantec Keypoint 4 (Medtronic Inc, Denmark) device was used for electrophysiological testing. All the operations were performed by the same examiner.

### Statistical Analyses

Intention-to-treat analyses were conducted by including all available observations in the analysis. Effective rate was based on our previously study, in according to the clinical practice guideline of Bell’s palsy [1] and a clinical practice guideline of facial nerve electrodiagnostic for patients with facial palsy[26]. The HB Grading of grade 3 or higher, or if CAMPs have a side difference of 30% or greater is considered abnormal. After laser acupuncture treatment, HB Grading lower than grade 2, or CAMP’s side difference lower than 30% is considered to have therapeutic effects.

A planned sample size of 84 randomized patients (42 assigned to PBMT group and 42 assigned to control group) were required, assuming 95% improvement rate (HB <  = 2) in the LA group, and 65% improvement rate in the control group, and provide 80% power. The test statistic used was the two-sided Fisher's Exact Test. The significance level of the test was 0.05. For the sample size calculation PASS 15.0 software (NCSS, Kaysville, UT) was used.

Statistical analysis was conducted from February 1, 2023, to February 28, 2023. SB Grading, latency of ENoG, EMG were described as means and SDs for normally distributed continuous variables, and as medians and interquartile ranges for nonnormally distributed continuous variables. Frequency with percentage was used to describe HB, ENoG and Blink Reflex.

Baseline characteristics are summarized according to facial paralysis and compared between participants with and without elevated depressive symptoms using the χ2 test, analysis of variance, or Mann–Whitney U test, as appropriate. All the analyses of patients with Bell’s palsy over 8 weeks were preformed based on the full analysis set. Missing primary outcome data and secondary visual acuity outcomes were imputed with Markov chain–Monte Carlo (MCMC) multiple imputation. We created 25 imputed data sets and pooled the results using the SAS statistical software version 9.4 (SAS Institute, Cary, NC).

Demographic data were analyzed by means of chi-square tests (× ^2^) or Fisher's exact tests, t-tests or non-parametric tests based on different data types. SB Grading, ENoG and EMG were all analyzed by Student t-tests or non-parametric tests, as appropriate. HB, ENoG and Blink Reflex were summarized with frequencies and percentages. Their distributions were assessed with chi-square tests or Fisher's exact tests.

We compared the proportions of abnormal Categorical variables results in the LA groups and control group using odd ratio (OR)with associated 95% CIs. All reported P values were two-sided and were declared statistically significant when less than 0.05.

## Results

**Fig. 2:**
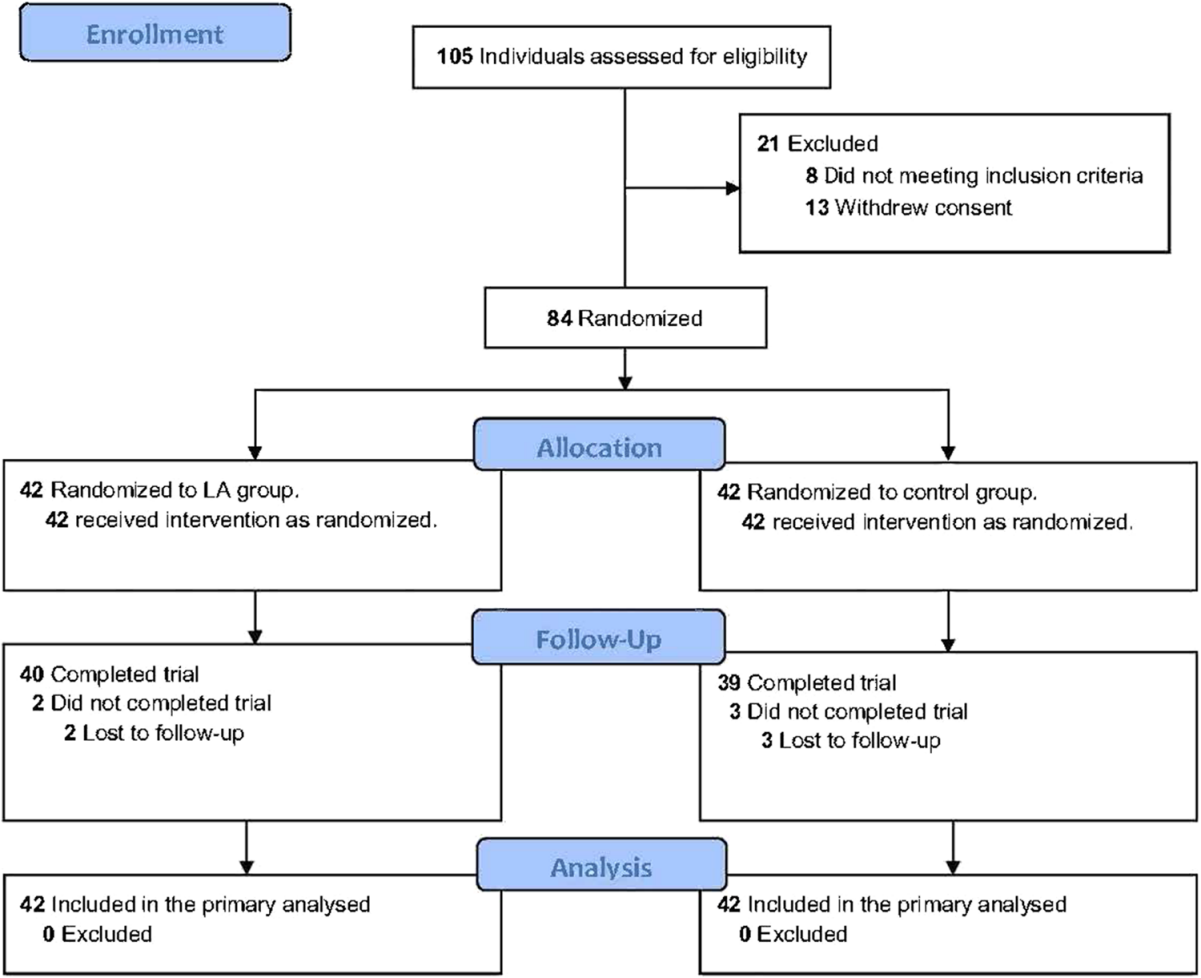
Study Flowchart

Between May 2021 and April 2023, 105 patients were screened for eligibility; 84 patients were included (42 in the control group and 42 in the LA group). Due to COVID-19, 5 participants (3 from the control group and 2 from the LA group) were unable to travel to the hospital. As a result, 79 participants were included in the analysis, with 39 in the control group and 40 in the LA group, as shown in the patient flow chart (Fig. 2). Patient- and treatment-related characteristics are presented in Table 1. The intention-to-treat population comprised all 84 patients in the LA group and control group (Table 2).

**Table 2:** Baseline Demographic Patient Characteristics

Characteristic	LA group	Control group
Age, mean (SD), year	45.43 (13.14)	41.67 (13.81)
Gender, No. (%)		
Female	26 (61.90)	31 (73.81)
Male	16 (38.10)	11 (26.19)
Body mass index, mean (SD)	25.15 (5.36)	26.49 (5.89)
Affected side of facial paralysis, No. (%)		
Right	21 (50.00)	27 (64.29)
Left	21 (50.00)	15 (35.71)
Duration of facial paralysis, mean (SD), month	14.05 (3.57)	14.36 (3.35)
Presence of hypertension, No. (%)		
Yes	3 (7.14)	6 (14.29)
No	39 (92.86)	36 (85.71)
Presence of diabetes, No. (%)		
Yes	6 (14.29)	0 (0)
No	36 (85.71)	42 (100.00)
Use of corticosteroid medications		
Yes	28 (66.67)	26 (61.90)
No	14 (33.33)	16 (38.10)
Use of Acupuncture		
Yes	18 (42.86)	22 (52.38)
No	24 (57.14)	20 (47.62)
Use of Exercise		
Yes	18 (42.86)	20 (47.62)
No	24 (57.14)	22 (52.38)

Abbreviations: LA, Laser Acupuncture

### Primary Outcome

Table 3 shows all the 84 participants (LA group 42 and control group 42) were abnormal HB results. After 12 weeks treatment, After 12 weeks, 11 abnormal HB results in LA group (OR, 232.83, 95% CI, 13.22 to 4100.92, P < 0.001), and 32 abnormal caloric test results in control group (OR, 27.46, 95% CI, 1.55 to 486.07, P < 0.001). The numbers of abnormal HB results in LA group had statistically significantly lower than those randomized to the control group at 12 weeks (OR, 0.11; 95% CI, 0.04–0.30; P < 0.001).

**Table 3:** Comparison of Categorical Variables Outcome Measures

	Baseline				Follow-up			
	LA group No./Total No. (%)	Control group No./Total No. (%)	P value	ORs (95% CI)	LA group No./Total No. (%)	Control group No./Total No. (%)	P value	ORs (95% CI)
HB 1	100(42/42)	100(42/42)	> 0.99	Not application	26.19(11/42) **	76.19(32/42) **	< .001	0.11 (0.04–0.30)
ENoG^2^								
Orbicularis oculi	100(42/42)	100(42/42)	> 0.99	Not application	11.90(5/42) **	66.67(28/42) **	< .001	0.08(0.02–0.21)
Frontalis muscle	97.62(41/42)	100(42/42)	> 0.99	0.33(0.01–8.22)	19.05(8/42) **	61.90(26/42) **	< .001	0.14(0.05–0.39)
Orbicularis oris	100(42/42)	100(42/42)	> 0.99	Not application	14.29(6/42) **	57.14(24/42) **	< .001	0.13(0.04–0.36)
Ala nasi muscle	100(42/42)	100(42/42)	> 0.99	Not application	14.29(6/42) **	75.61(31/42) *	< .001	0.06(0.02–0.18)
Blink reflex 3								
R1	95.24(40/42)	97.62(41/42)	> 0.99	0.49(0.04–5.59)	4.76(2/42) **	59.52(25/42) **	< .001	0.03(0.01–0.16)
R2	100(42/42)	100(42/42)	> 0.99	Not application	2.38(1/42) **	40.48(17/42) **	< .001	0.04(0.004–0.29)
R2 differences	71.63(30/42)	61.90(26/42)	0.35	1.54(0.62–3.84)	9.52(4/42) **	40.48(17/42)	< .001	0.15(0.05–0.51)

Abbreviations: LA, Laser Acupuncture; HB, House–Brackmann facial nerve grading system; ENoG, Electroneurography; NA, not available; ORs, Odds ratios

^*^p < 0.05 and **p < 0.001 comparison between groups by 2‐sample t test (2‐tailed for baseline, superiority for change from baseline at follow‐ups

1 The HB is based on a 6-grade score that offers a gross evaluation of facial motor function. The prognoses of patients with grade 3 or higher were considered poor. All the HB grading were assessed by the same medical chief physician

2 The testing of ENoG involves recording the CMAPs of the mimetic muscles, including Orbicularis oculi, Frontalis muscle, Orbicularis oris and Musculus levator superioris alaeque nasi, the amplitude of the CMAPs obtained was measured, and the affected side and the normal side were compared. A percentage of degenerated nerve fibers is calculated. A side difference of 30% or bigger is considered pathologic in our study

3 In Blink Reflex testing, two responses, R1 and R2, are analyzed. R1 is the fast ipsilateral response of the orbicularis oculi muscle with a latency of about 10–12 ms. The second bilateral response R2 has a latency of about 30–41 ms. The R1 latency higher than 12 ms, or the The R2 latency higher than 41 ms is considered pathologic. The R2 latency differences between both sides higher than 8 ms is considered pathologic

Table 3 also indicates that all participants were abnormal ENoG results, orbicularis oculi, orbicularis oris and ala nasi muscle (LA group 42 and control group 42) were. 41 in LA group and 42 in control group were abnormal Frontalis muscle ENoG results.

After 12 weeks, 5 abnormal orbicularis oculi ENoG results in LA group (OR, 579.54, 95% CI, 31.00 to 10,833.69, P < 0.001), with 28 in control group (OR, 43.25, 95% CI, 2.48 to 754.27, P < 0.001). 8 abnormal Frontalis muscle ENoG results in LA group (OR, 174.25, 95% CI, 20.75 to 1463.34, P < 0.001), with 26 in control group (OR, 52.92, 95% CI, 3.05 to 919.54, P < 0.001). 6 abnormal Orbicularis oris. ENoG results in LA group (OR, 451.15, 95% CI, 24.545 to 8292.48, P < 0.001), with 24 in control group (OR, 64.18, 95% CI, 3.70 to 1112.56, P < 0.001). 6 abnormal Ala nasi muscle ENoG results in LA group (OR, 477.31, 95% CI, 26.00 to 8764.01, P < 0.001), with 31 in control group (OR, 52.92, 95% CI, 3.05 to 919.54, P < 0.001).

All the numbers of abnormal ENoG results in LA group had statistically significantly lower than those randomized to the control group at 12 weeks, orbicularis oculi (-0.29; 95% CI, -0.53 to -0.04; P = 0.03), Frontalis muscle (-0.30; 95% CI, -0.42 to -0.18; P < 0.001), Orbicularis oris (-0.29; 95% CI, -0.53 to -0.05; P = 0.02), Ala nasi muscle (-0.29; 95% CI, -0.47 to -0.11; P < 0.01).

**Fig. 3:**
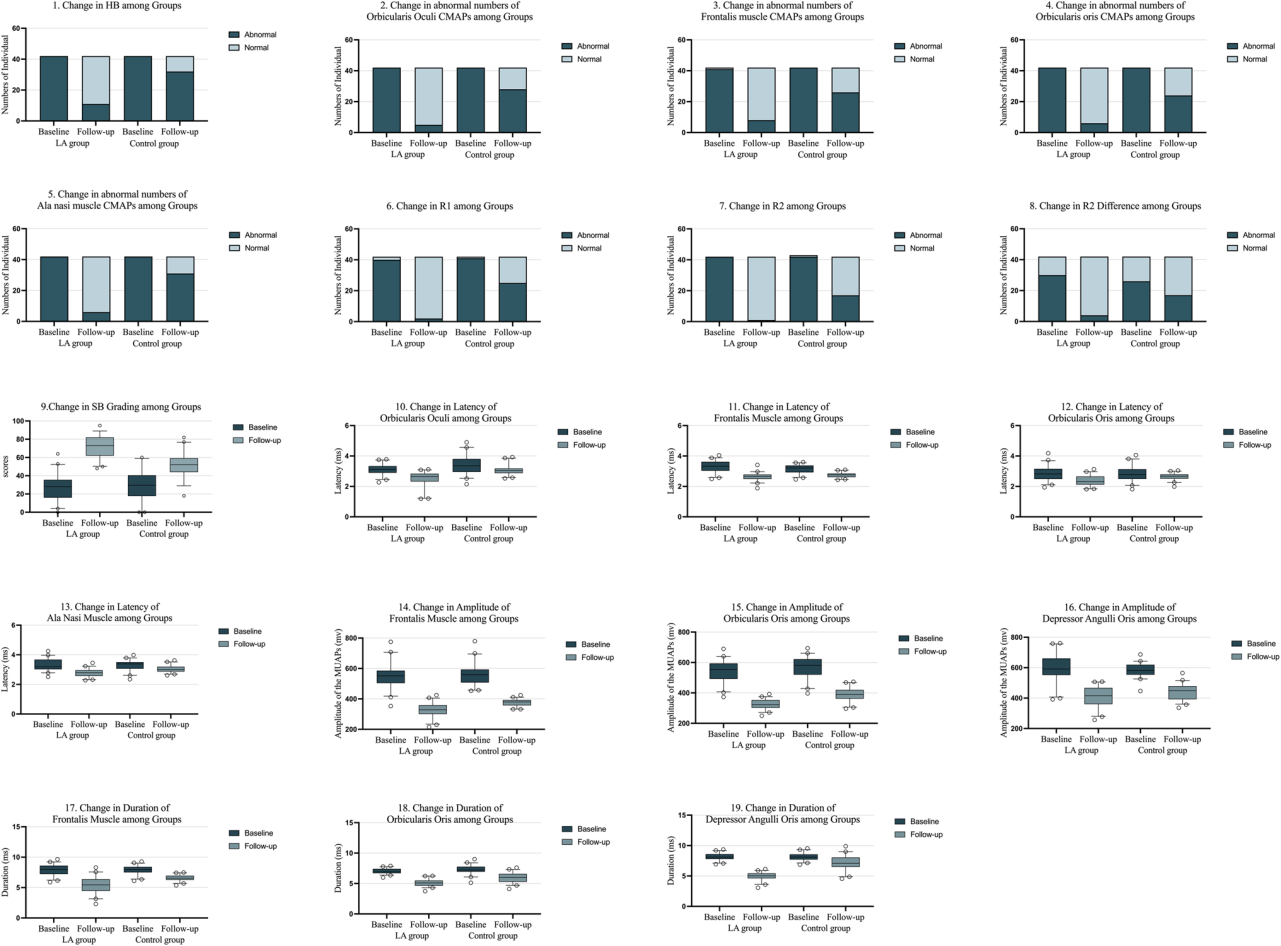
Outcome Measures in the LA group and Control Groups. Abbreviation. LA: Laser acupuncture. HB: House–Brackmann grading system, CMAPs: compound muscle action potentials. SB Grading: Sunnybrook Facial Grading Scale. MUAPs: motor unit action potentials

All the numbers of abnormal ENoG results in LA group had statistically significantly lower than those randomized to the control group at 12 weeks, orbicularis oculi (OR,0.08; 95% CI, 0.02–0.21; P < 0.001), Frontalis muscle (OR,0.14; 95% CI, 0.05–0.39; P < 0.001), Orbicularis oris (OR,0.13; 95% CI, 0.04–0.36; P < 0.001), Ala nasi muscle (OR,0.06; 95% CI, 0.02–0.18; P < 0.001) (Fig. 3).

### Secondary Outcomes

Compared with baseline, Table 4 shows that SB grading was 44.62 points lower after treatment in the LA group (95% CI, 2.77 to 4.30; P < 0.01) and 22.64 points lower in the control group (95% CI, 3.09 to 4.79; P < 0.01). Participants in the LA group had statistically significant higher scores compared with those in the control group after treatment (20.26; 95% CI, 14.69 to 25.83; P < 0.01) (Fig. 3).

**Table 4:** Comparison of Continuous Variables Outcome Measure

	Baseline				Follow-up			
	LA group	Control group	Mean difference	P value	LA group	Control group	Mean difference ^a^	P value
SB Grading 1	27.29 (14.58)	29.00 (16.47)	-1.71(-8.47 to 5.04)	0.61	71.90 (11.98)	51.64 (13.63)	20.26 (14.69 to 25.83)	< .001
Latency (ms) of ENoG 2								
Orbicularis oculi	3.30(0.26)	3.45(0.62)	-0.15 (-0.36 to 0.05)	0.14	2.47(0.52)	3.08(0.32)	-0.61 (-0.43 to -0.09)	< .001
Frontalis muscle	3.23 (0.37)	3.14(0.29)	0.09 (-0.05 to 0.24)	0.21	2.63(0.25)	2.75(0.17)	-0.12 (-0.21 to -0.03)	0.01
Orbicularis oris	2.84(0.46)	2.83(0.51)	0.01 (-0.21 to 0.22)	0.96	2.36(0.34)	2.65(0.21)	-0.28 (-0.41 to -0.16)	< .001
Ala nasi muscle	3.32(0.40)	3.29(0.36)	0.02 (-0.14 to 0.19)	0.74	2.77(0.27)	3.04(0.24)	-0.26 (-0.38 to -0.16)	< .001
Amplitude of the MUAPs (mv) 2								
Frontalis muscle	551.53(79.99)	558.70(70.42)	-21.79 (-52.21 to 8.62)	0.16	328.26(43.94)	375.32(22.08)	-64.23 (-80.89 to -47.56)	< .001
Orbicularis oris	544.11(68.77)	565.90(50.07)	14.02 (-16.55 to 44.59)	0.36	325.30(32.13)	389.54(43.76)	-29.82 (-55.03 to -4.62)	0.02
Depressor angulli oris	600.31(89.07)	586.29(44.56)	-7.17 (-39.87 to 25.55)	0.66	409.97(66.14)	439.79(48.66)	-47.06 (-62.15 to -31.97)	< .001
Duration (ms) of EMG 2								
Frontalis muscle	7.93 (0.85)	7.89(0.73)	0.05(-0.21 to 0.30)	0.72	5.43(1.33)	6.57(0.48)	-1.71(-8.47 to 5.04)	< .001
Orbicularis oris	7.11 (0.37)	7.30(0.71)	-0.19 (-0.44 to 0.05)	0.12	5.08(0.57)	5.96(0.81)	-2.16 (-2.58 to -1.74)	< .001
Depressor angulli oris	8.17(0.55)	8.13(0.62)	0.04(-0.30 to 0.38)	0.82	4.94(0.65)	7.09(1.22)	-1.14 (-1.58 to 0.71)	< .001

Abbreviations: LA, Laser Acupuncture; SB Grading, Sunnybrook facial grading system; CMAPs, compound muscle action potentials; MUAPs, motor unit action potentials; ENOG, Electroneuronography; EMG, Electromyography

1, Facial paralysis symptoms were measured using SB grading (range, 0–100; Lower scores are equivalent to greater severity of facial paralysis symptoms)

2, Electrophysiological examinations were measured using Electroneurography and Electromyography. The testing of ENoG involves recording the CMAP of muscles, and EMG measures that facial nerve function by recording MUAPs

After treatment, latency of ENoG were lower than the means in baseline, orbicularis oculi (0.83; 95% CI, 0.39 to 2.47; *P* < 0.001), frontalis muscle (0.60; 95% CI, 0.27 to 0.41; *P* < 0.001), orbicularis oris (0.47; 95% CI, 0.48 to 0.75; *P* < 0.001), and ala nasi muscle (0.54; 95% CI, 0.35 to 0.54; *P* < 0.001). In control group, latency of ENoG were lower than the means in baseline, orbicularis oculi (0.37; 95% CI, 0.51 to 0.78; P < 0.001), frontalis muscle (0.39; 95% CI, 0.30 to 0.47; P < 0.001), orbicularis oris (0.19; 95% CI, 0.41 to 0.64; P = 0.02), and ala nasi muscle (0.25; 95% CI, 0.33 to 0.52; P < 0.001). Table 4 also demonstrates that the latency by ENoG showed statistically significant differences compared with the control group for orbicularis oculi (-0.61; 95% CI, -0.43 to -0.09; P < 0.001), frontalis muscle (-0.12; 95% CI, -0.21 to -0.03; P < 0.01), orbicularis oris (-0.28; 95% CI, -0.41 to -0.16; P < 0.001), and ala nasi muscle (-0.26; 95% CI, -0.38 to -0.16; P < 0.001) (Fig. 3).

In LA group, MUAPs were lower than the means in baseline, frontalis muscle (223.30; 95% CI, 79.68 to 123.50; P < 0.001), orbicularis oris (218.80; 95% CI, 64.22 to 99.53; P < 0.001), depressor angulli oris (190.30; 95% CI, 94.58 to 146.60; P < 0.001). Duration was lower than the means in baseline, frontalis muscle (2.50; 95% CI, 0.42 to 0.64; P < 0.001), orbicularis oris (2.03; 95% CI, 0.48 to 0.74; P < 0.001), depressor angulli oris (3.24; 95% CI, 0.70 to 1.09; P < 0.001) after treatment.

Compared with baseline in control group, Table 4 shows that MUAPs were lower than the means in baseline, frontalis muscle (183.40; 95% CI, 60.93 to 94.43; P < 0.001), orbicularis oris (176.40; 95% CI, 69.04 to 107.00; P < 0.001), depressor angulli oris (146.50; 95% CI, 46.90 to 72.68; P < 0.001). Durations were lower than the means in baseline, frontalis muscle (1.32; 95% CI, 0.72 to 1.11; P < 0.001), orbicularis oris (1.34; 95% CI, 0.83 to 1.29; P < 0.001), orbicularis oris, depressor angulli oris (1.03; 95% CI, 1.09 to 1.69; P < 0.001).

All amplitudes of MUAPs and durations by EMG showed statistically significant differences compared with the control group after treatment. For the frontalis muscle, the amplitude of MUAPs was -64.23 (95% CI, -80.89 to -47.56; P < 0.001) and duration was -1.18 (95% CI, -1.49 to -0.87; P < 0.001). For orbicularis oris, amplitude of MUAPs was -29.82 (95% CI, -55.03 to -4.62; P = 0.02) and duration was -0.57 (95% CI, -0.94 to -0.20; P < 0.001). For depressor angulli oris, amplitude of MUAPs was -47.06 (95% CI, -62.15 to -31.97; P < 0.001) and duration was -2.21 (95% CI, -2.69 to -1.72; P < 0.001) (Fig. 3).

Table 3 also indicates that pathologic numbers of R1 (LA group 40 and control group 41), R2 (LA group 42 and control group 42) and R2 latency differences (LA group 30 and control group 26) were abnormal BR results.

After 12 weeks, 2 abnormal R1 results in LA group (OR, 400, 95% CI, 53.68 to 2980.45, P < 0.001), with 25 in control group (OR, 27.88, 95% CI, 3.49 to 222.54, P < 0.001).

1 abnormal R2 results in LA group (OR, 2351.67, 95% CI, 93.11 to 59,393.60, P < 0.001), with 17 in control group (OR, 123.86, 95% CI, 7.14 to 2149.10, P < 0.001). 4 abnormal R2 latency differences results in LA group (OR, 23.75, 95% CI, 6.95 to 81.15, P < 0.001), with 17 in control group (OR, 2.38, 95% CI, 1.00 to 5.73, P = 0.049).

All the numbers of abnormal BR results in LA group had statistically significantly lower than those randomized to the control group at 12 weeks, R1 (OR, 0.03; 95% CI, 0.01–0.16; P < 0.001), R2 (OR, 0.04; 95% CI, 0.004–0.29; P < 0.001), and R2 latency differences (OR, 0.15; 95% CI, 0.05–0.51; P < 0.001).

## Discussion

Currently, there is very few treatments for Bell’s palsy over 8 weeks other than recover naturally [1]. Acupuncture plays an important role in regulating “Qi and blood” in treating Bell’s Palsy, and the scientific mechanisms of acupuncture have been proven to be through multiple targets and multiple systems [20, 31, 32] and laser acupuncture and acupuncture show to have at least similar efficacy [33–35]. Therefore, our intervention included PBMT in affected side, and acupoints in abdomen, upper extremities and lower extremities in this study. In the treatment of facial paralysis, PBMT often involves scanning the areas covered by the facial nerve. In this study, the acupuncture points and the facial areas scanned by PBMT are similar. Therefore, the therapeutic effects observed in this study can be attributed to a combined effect of both PBMT and laser acupuncture [16].

The evaluation of facial nerve damage and prediction of the prognosis are important to patients with facial paralysis. We used objective and subjective facial nerve damage measures, to provide a comprehensive assessment.

After treatment, the results of HB and SB Grading indicated that laser acupuncture can improve the symptoms. The amplitude and latency of CMAPs reflects the degree of facial nerve degeneration on the affected side. As patients with facial palsy, the amplitude of CMAPs decreased and the latency increased. After treatment, the amplitude of CMAPs of Orbicularis oculi, frontalis muscle, orbicularis oris and ala nasi muscle increased. And the latency of orbicularis oculi, frontalis muscle, and orbicularis oris decreased after treatment.

EMG analyzes the facial MUAPs, which are the spikes in electrical activity generated when a motor unit fires. The duration of MUAPs is increased in patients with axonotmesis or neurotmesis. In our study, the amplitude of and duration of MUAPs decreased after treatment.

The Blink Reflex is mediated through the trigeminal nerve, progressing to the trigeminal nucleus, followed by the facial nerve nucleus and ultimately the facial nerve. After laser acupuncture treatment, there's a reduction in the counts of abnormal R1, R2, and the R2 differences on both sides. Combined with subjective rating scales and electrophysiological examinations, we suggest that laser acupuncture can improve the facial nerve function and promote rehabilitation.

Pasquale et al. used Class IV laser device to treat facial paralysis. They treated 14 Bell’s palsy over 8 weeks with laser device (808 nm, 1 W). At the end of the trails, 11 of 14 patients’ HB grading decreased to grade I [18]. Ton et al. treated a 52-year-old male with facial paralysis lasted for 12 years with laser (810 nm, 200 mW). After 30 sessions treatment, the symptoms were improved [19]. Alayat et al. evaluated laser therapy’s effectiveness on 60 patients (three groups of 20 patients each) with idiopathic Bell's palsy, significant recovery improvement was noted as measured by the HB and Facial Disability Index over 6 weeks [16].

Some limitations in our study should be noted. Our study only collected baseline and follow-up outcome measure. Future studies should incorporate a longer follow-up period. Additionally, we did not include iatrogenic facial paralysis, trauma, or bilateral facial paralysis. In the future, we will recruit facial paralysis with multifactorial etiology.

Stimulating acupoints on the limbs and trunk, such as LI4 (He Gu), ST25 (Tian Shu) and ST36 (Zu San Li), can treat facial paralysis. According to traditional Chinese medicine (TCM) theory, which attributes the effect to meridian regulation. However, since there is limited research on lasers' impact on TCM meridians, we currently cannot explain the mechanism behind treating facial paralysis through acupoints on the limbs and trunk. Future interdisciplinary research is required to elucidate this treatment mechanism.

## Conclusions

The findings of this randomized controlled trial, single-center study suggest that laser acupuncture and photobiomodulation therapy relieve symptoms for patients with Bell’s palsy over 8 weeks.
